# A High-Impedance Grounding Fault Identification Method for Mining Cables in Non-Effectively Grounded Systems of Coal Mine Power Grids Based on Steady-State Impedance Analysis–Holmes–Duffing

**DOI:** 10.3390/s25216675

**Published:** 2025-11-01

**Authors:** Chen Feng, Long Ni, Yunfeng Lan, Huizhong Zheng, Xiangjun Zeng

**Affiliations:** 1 The College of Electrical Engineering and Automation, Shandong University of Science and Technology, Qingdao 266590, China; nilong184486@sdust.edu.cn (L.N.); lanyunfeng02@163.com (Y.L.); 19963296981@163.com (H.Z.); 2 College of Electrical Engineering and New Energy, China Three Gorges University, Yichang 443002, China; sxdxzxj@ctgu.edu.cn

**Keywords:** fault line selection, steady-state impedance analysis, Holmes–Duffing, mining cable, non-effectively grounded system

## Abstract

**Highlights:**

**What are the main findings?**

- This paper proposes a steady-state impedance analysis (SSIA) method. By utilizing the stability of steady-state signals in the distribution network, fault characteristic analysis becomes more convenient.
- A Holmes–Duffing oscillator-based small-signal detection method is proposed, which enables effective measurement of weak signals.

**What is the implication of the main finding?**

- By using the difference in zero-sequence current of each line before and after a fault as the fault criterion, fault line selection becomes more accurate.
- Image processing is applied to the phase diagrams output by the oscillator. Case studies demonstrate that the proposed method improves the measurement capability for fault line selection. Fault identification is not affected by measurement accuracy, resulting in more precise fault detection.

**Abstract:**

In coal mine non-solidly grounded systems, high-impedance faults generate minimal zero-sequence currents with obscured characteristics and strong interference, complicating faulted line identification. Existing methods rarely address three-phase imbalance and variable cable parameters, causing selection errors. To this end, a method for identifying the non-effective ground fault routing of mining cables based on Steady-State Impedance Analysis (SSIA) and Holmes–Duffing oscillator small-signal detection is proposed. Firstly, based on SSIA, the mapping relationship that the phase of the zero-sequence current variation in the faulted line is the same as the phase of its voltage relative to the faulted ground is derived before and after the occurrence of the fault. Meanwhile, identifiable differences exist in both phase and amplitude of the zero-sequence current change in faulty lines compared to non-faulty lines before and after fault occurrence. This is used as the criterion for high-impedance ground fault line selection. In the mining environment, zero-sequence current variations are characterized as weak signals, which poses significant challenges for detection. Thus, a Holmes–Duffing oscillator weak signal detection method is proposed. Based on chaotic principles, accurate line selection is achieved by diagnosing chaotic states in oscillator-generated phase trajectories. A specific mine grid simulation via MATLAB/Simulink 2023b validates the method’s efficacy and applicability.

## 1. Introduction

Coal mine 10 kV and 6 kV power grids generally employ non-effectively grounded systems, either with ungrounded neutrals [1] or neutrals grounded via arc-suppression coils [2]. When high-impedance ground faults occur in cables under this grounding configuration, fault characteristics are indistinct. Consequently, achieving accurate faulty line identification has remained a persistent research focus in the electrical engineering domain [3,4,5,6,7,8]. Currently, scholars domestically and internationally have conducted extensive research, proposing diverse faulty line identification approaches. Among these, methods for non-effectively grounded systems primarily proceed from two aspects: steady-state characteristics [9,10,11,12,13,14] and transient characteristics [15,16,17]. Refs. [9,10,11] employed the zero-sequence current characteristic comparison method, identifying the faulty line by comparing the variations in these characteristics across all lines. Ref. [12] employed a group amplitude–phase comparison method for zero-sequence currents. Three lines possessing the largest magnitudes are selected. Phases of these three lines are then contrasted to determine the faulted line. While all the cited methods utilize fault signatures derived from zero-sequence current, they share a common limitation: this approach demands high identification accuracy and is prone to misjudgment due to external disturbances. Ref. [13] accomplished high-impedance ground fault line selection in flexibly grounded systems. This is realized by comparing zero-sequence measured impedances across all lines. Ref. [14] performed EMD decomposition on steady-state current signals. Fifth harmonic components of line currents are input into Duffing oscillator systems for line selection determination. The unsupervised approach of directly using extracted signals as input for the Duffing oscillator system demands further validation regarding the interpretability and adaptability of its outcomes. Ref. [15] applied discrete wavelet transform to extract zero-sequence current features. Normalization processing is executed. Signals from different frequency bands serve as characteristic quantities. A PSO-SVM fault diagnosis model is trained to accomplish fault diagnosis. Ref. [16] leveraged the fault location principle of transient components. Components in zero-sequence networks are collected. Precise location is achieved through a grouping comparison scheme. Ref. [17] introduced a traveling wave method based on a current waveform similarity comparison. Transient traveling wave information is fully exploited. Among these methods, electrical signals in steady-state characteristics—such as zero-sequence voltage, zero-sequence current, and three-phase voltages as state quantities—exhibit greater stability [18]. Their signal characteristics are more readily extractable. Consequently, this facilitates faulty line identification implementation. However, the aforementioned studies also share the following two common issues: First, diverse line faults (e.g., metallic/high-resistance grounding) exhibit distinct characteristics. Simultaneously addressing load imbalance and cable parameter variations reduces criterion applicability.

Second, steady-state zero-sequence current methods face inherent limitations. Studies confirm that these manifest as minute signals during high-impedance faults, hindering practical deployment.

To address these challenges, this paper advances a faulty line identification approach for non-effectively grounded faults in mining cables based on SSIA–Holmes–Duffing weak signal detection. The criterion derived from the SSIA provides a theoretical foundation for subsequent fault line selection, thereby addressing the interpretability issue of unsupervised fault identification methods. The variation in zero-sequence current from each line is employed as the input to the Duffing oscillator system, to mitigate the issue of high yet sometimes insufficient accuracy in methods that rely solely on zero-sequence current for fault line selection. The Holmes–Duffing oscillator is employed for detecting zero-sequence current variations, solving the practical problem of these characteristic signals being too weak for reliable acquisition. Furthermore, validation was conducted under scenarios of three-phase unbalance and faults on different cables to demonstrate the adaptability of the proposed method. The primary contributions of this work are as follows:

1. A fault line selection criterion based on the steady-state impedance analysis method is proposed. Pre/post-fault, the faulted line’s zero-sequence current change quantity exhibits distinct phase characteristics versus that of non-faulted lines. Within coal mine distribution network impedance models, analysis under ungrounded/arc-suppression-coil grounding modes confirms the following: during high-resistance faults, steady-state impedance and zero-sequence current features derive differential phase/amplitude in faulted lines’ change quantities. This enables accurate line selection in non-solidly grounded systems.
2. A small-signal detection method for the Holmes–Duffing oscillator system is proposed. During high-resistance grounding faults in coal mine non-effectively grounded systems, zero-sequence current change quantities manifest as minute signals (μA–mA range), which are challenging for practical detection. This work extracts these signals from faulted lines as Holmes–Duffing oscillator inputs, leveraging the high micro-signal sensitivity for feature extraction. Subsequent chaos principle analysis generates line-specific phase trajectories, examines chaos–periodic state relationships with external driving forces, and compares output phase diagrams to achieve precise fault line selection.

The remainder of this paper is organized as follows:

Section 2 establishes an equivalent model of the coal mine distribution network based on an actual mine. It discusses the derivation of criteria for faulty line selection using the steady-state impedance analysis method under both ungrounded neutral and neutral grounded via arc-suppression-coil conditions. Additionally, it analyzes the adaptability of the proposed criteria under scenarios of three-phase load unbalance and different cable parameters. Section 3 primarily introduces the principles of the Holmes–Duffing oscillator system and details the parameter settings for this system. Section 4 presents the algorithm flowchart of this paper. Section 5 mainly conducts an analysis of the effectiveness of the criteria proposed in Section 2 under the working conditions discussed earlier, as well as an analysis of the effectiveness of the weak signal detection method based on the Holmes–Duffing oscillator system under the discussed conditions. Section 6 summarizes the research presented in this paper and suggests directions for future research.

## 2. High-Impedance Grounding Fault Line Selection Criteria Based on Steady-State Impedance Analysis

In coal mine distribution networks, high-voltage main supply lines are commonly incorporated. These transmit power from substations to key equipment and areas in mining zones. Incoming lines of coal mine substations pass through primary transformers to reach medium–low voltages of 6 kV or 10 kV. The 6 kV and 10 kV power supply systems comprise multiple supply zones. At the 10 kV side of the 110/10 kV substation transformers in coal mines, neutral grounding methods generally adopt non-effectively grounded configurations. Specifically, these encompass ungrounded neutral methods and neutral grounding via arc-suppression coils. Circuit analysis for both methods will follow [19].

### 2.1. Steady-State Impedance Analysis of High-Impedance Grounding Faults in Ungrounded Neutral-Point Systems

**Figure 1:**
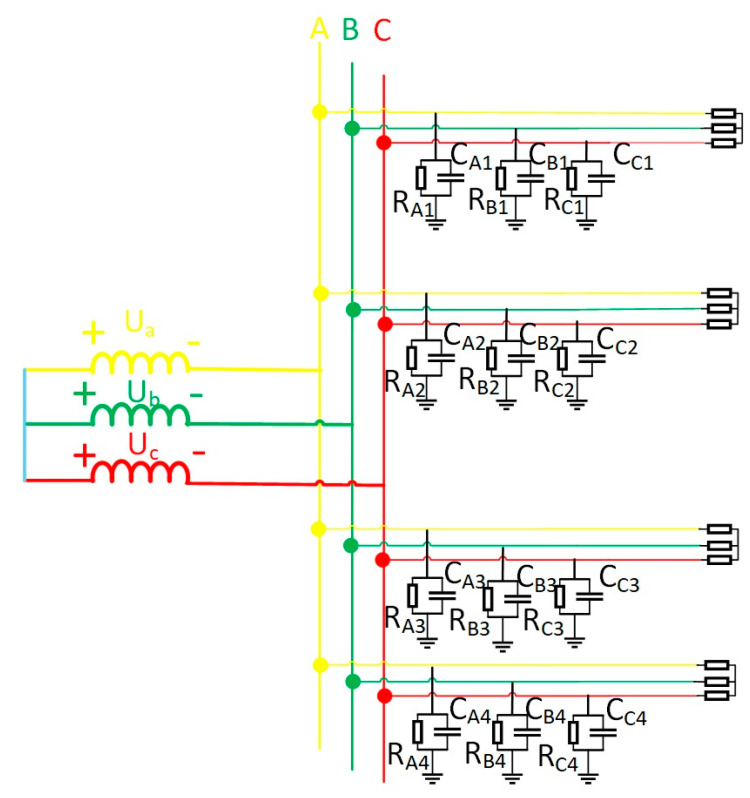
Equivalent model of coal mine power grid.

During steady-state single-phase grounding fault analysis, line admittance effects are negligible. Line resistance and inductive reactance magnitudes fall significantly below circuit capacitive reactance, permitting their omission in equivalent circuits [20]. This paper establishes a coal mine distribution network model (Figure 1) reflecting actual 10 kV underground configurations. The neutral point remains ungrounded, with a single busbar supplying four feeders. Asymmetrical faults—single-phase grounding, two-phase shorts, and phase breaks—represent unbalanced conditions. These induce unequal three-phase impedances, resulting in divergent RMS voltage/current values and phase shifts, thus warranting symmetrical component analysis for fault examination [21].

**Figure 2:**
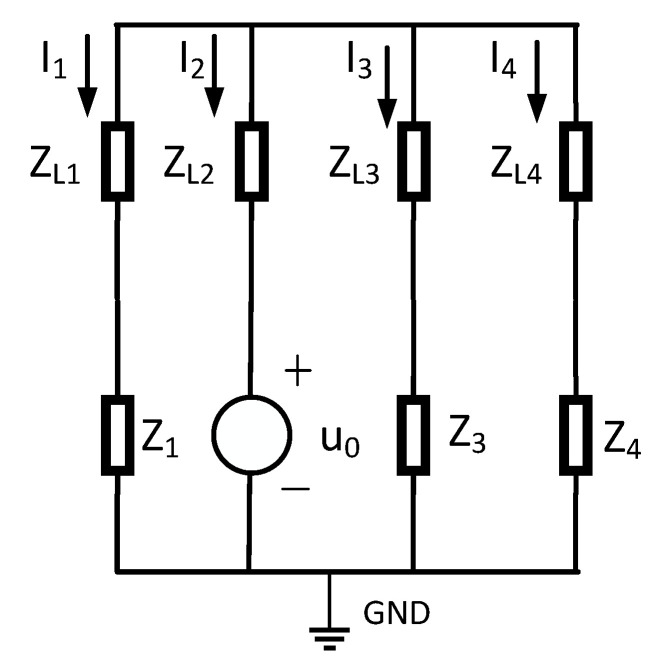
Zero-sequence network of coal power and insulation resistance and reactance parallel structure.

In Figure 1, four cables operate normally. Assume a single-phase high-impedance ground fault occurs on the second cable. This generates zero-sequence current in the system. The equivalent zero-sequence network is depicted in Figure 2. When a fault emerges on the second line, a voltage source identical in magnitude and phase to the zero-sequence voltage is introduced at the fault point within the zero-sequence network. This voltage source serves as the equivalent circuit. For coal mine power grids, the longest cable supply line generally does not exceed 3 km. In short-distance cables, employing π-type or T-type equivalent circuit parameters exerts negligible influence on steady-state analysis outcomes. This work adopts T-type equivalent circuits for lines in the zero-sequence network. In the figure, $Z_{L1},\;Z_{L2},\;Z_{L3},\;Z_{L4}$ are the zero-sequence impedances of the four cable lines, $Z_{1},\;Z_{3},\;Z_{4}$ are the insulation impedance of the first line, the third line, and the fourth line, respectively; the end of the line is connected with the load. ${\dot{I}}_{1},\;{\dot{I}}_{2},\;{\dot{I}}_{3},\;{\dot{I}}_{4}$ in the figure are the zero-sequence currents of the four lines.

**Figure 3:**
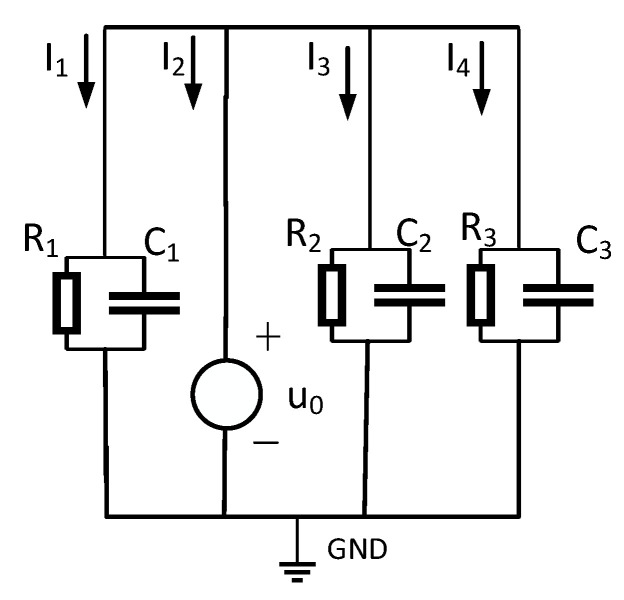
Simplified map of zero-sequence network.

In Figure 3, the insulation impedance of the cable is the sum of the three-phase insulation impedances, where the insulation impedance consists of the parallel combination of the insulation resistance and the distributed capacitance $Z_{n}=\frac{R_{n}}{j\omega C_{n}R_{n}+1}$. Moreover, the cable insulation impedance is much greater than the line’s zero-sequence impedance, i.e., $Z_{n}>>Z_{Ln}$. Therefore, Figure 2 can be simplified, ignoring the zero-sequence impedance of the line and showing the insulation impedance. The simplified zero-sequence network diagram is shown in Figure 3.

The relationship between the zero-sequence current and the zero-sequence voltage of the non-fault phase line is as follows:

(1) $${\dot{I}}_{n}=\frac{{\dot{U}}_{0}(j\omega C_{n}R_{n}+1)}{R_{n}}$$

${\dot{I}}_{n}$(n = 1,3,4) is the zero-sequence current of the non-faulty line.

**Figure 4:**
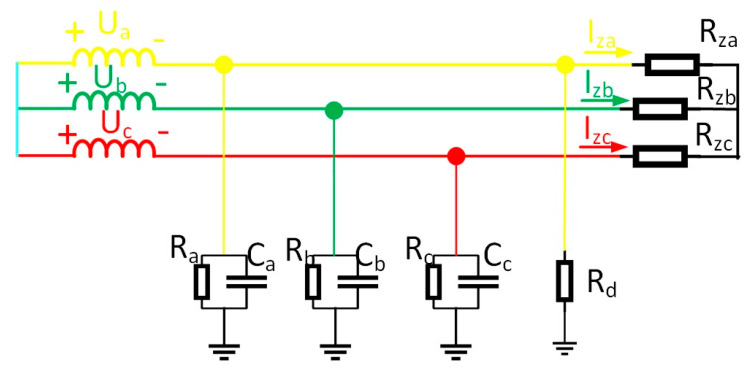
Equivalent operational circuit for faulty lines.

For faulted lines, Equation (1)’s zero-sequence current–voltage relationship fails. This work thus analyzes single-phase grounding faults, examining fault-line zero-sequence characteristics. The equivalent zero-sequence network appears in Figure 4. Assuming phase-A grounding faults—where metallic/high-resistance faults represent special cases—different fault types are simulated via grounding resistance adjustment. High-resistance values denote single-phase high-impedance faults. In the figure, ${\dot{U}}_{a},\;{\dot{U}}_{b},\;{\dot{U}}_{c}$ represent the phase voltage of the three-phase power supply, $R_{a},\;R_{b},\;R_{c}$ represent the insulation resistance to ground for the three phases, $C_{a},\;C_{b},\;C_{c}$ represent the distributed capacitance to ground for the three phases, and $R_{d}$ represents the equivalent resistance value of the faulted line. Based on the three-phase characteristics of the line, the three-phase insulation impedance and three-phase distributed capacitance of the cable are all equal, i.e., $R_{a}=R_{b}=R_{c}$, $C_{a}=C_{b}=C_{c}$ [22].

According to Kirchhoff’s current law, the zero-sequence current of a fault line is as follows:

(2) $${\dot{I}}_{i}=\frac{\left({\dot{U}}_{a}+{\dot{U}}_{0}\right)}{Z_{a}//R_{d}}+\frac{\left({\dot{U}}_{b}+{\dot{U}}_{0}\right)}{Z_{b}}+\frac{\left({\dot{U}}_{c}+{\dot{U}}_{0}\right)}{Z_{c}}$$

In Equation (2), the following values apply: $Z_{a}=\frac{R_{a}}{j\omega C_{a}R_{a}+1}$, $Z_{b}=\frac{R_{b}}{j\omega C_{b}R_{b}+1}$, $Z_{c}=\frac{R_{c}}{j\omega C_{c}R_{c}+1}$, and according to symmetry, $Z_{a}=Z_{b}=Z_{c}$. For the convenience of calculation simplification, let $Z_{a}=Z_{b}=Z_{c}=Z$, and the sum of the three-phase voltages is always zero, i.e., ${\dot{U}}_{a}+{\dot{U}}_{b}+{\dot{U}}_{c}=0$; substituting these conditions into Equation (2) simplifies to the following:

(3) $${\dot{I}}_{i}=\frac{3{\dot{U}}_{0}}{Z}+\frac{\left({\dot{U}}_{a}+{\dot{U}}_{0}\right)}{R_{d}}$$

**Figure 5:**
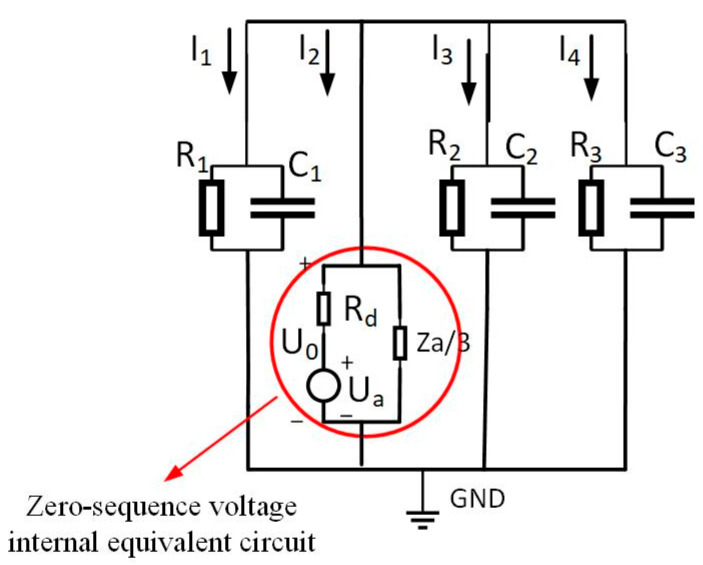
Zero-sequence equivalent circuit of a single-phase ground-faulted line.

As per Equation (3), zero-sequence voltage originates from the collective action of grounding impedance $R_{d}$, insulation impedance $Z_{a}$, and phase voltage ${\dot{U}}_{a}$ of the faulted phase. Based on the formula, internal equivalence for the zero-sequence voltage in Figure 3 is achievable. The equivalent circuit is illustrated in Figure 5.

According to Kirchhoff’s current law, the following is obtained:

(4) $${\dot{I}}_{1}+{\dot{I}}_{2}+{\dot{I}}_{3}+{\dot{I}}_{4}=0$$

Using Ohm’s law to express the currents in Equation (4) in terms of voltage and impedance, the relationship between the zero-sequence voltage and phase voltage of the system at fault is as follows:

(5) $${\dot{U}}_{0}=-\frac{{\dot{U}}_{a}}{(j\omega C_{\Sigma }+\frac{1}{R_{\Sigma }})R_{d}}=-\frac{{\dot{U}}_{a}}{\sqrt{\frac{{R_{d}}^{2}}{{R_{\Sigma }}^{2}}+\omega^{2}C_{\Sigma }^{2}R_{d}^{2}}}e^{-j\vartheta }$$

In the formula:$\frac{1}{R_{\Sigma }}=\frac{1}{R_{1}}+\frac{1}{R_{3}}+\frac{1}{R_{4}}+\frac{3}{R_{a}}$, $C_{\Sigma }=C_{1}+C_{3}+C_{4}+3C_{a}$, $\vartheta =\arctan(j\omega C_{\Sigma }R_{\Sigma })$.

Thus, the phase voltage of the faulted phase leads the zero-sequence voltage by (90°–180°) degrees [23]. Integrating zero-sequence current analysis, prior to single-phase grounding fault occurrences in the system, grounding resistance is absent (i.e., $R_{d}=0$). Substituting this condition into Equation (2) yields the pre-fault system zero-sequence current as ${{\dot{I}}^{\prime }}_{i}=\frac{3{{\dot{U}}^{\prime }}_{0}}{Z}$. The post-fault system zero-sequence voltage follows Equation (3). Subtraction of the pre-fault from the post-fault system zero-sequence currents yields the zero-sequence current variation, as Equation (6) demonstrates:

(6) $$\Delta {\dot{I}}_{i}=\frac{3({\dot{U}}_{0}-\dot{U_{0}^{\prime }})}{Z}+\frac{\left({\dot{U}}_{a}+{\dot{U}}_{0}\right)}{R_{d}}$$

In Equation (6), $\dot{U_{0}^{\prime }}$ and ${\dot{U}}_{0}$ are the zero-sequence voltage before and after the fault, respectively. And $\left|{\dot{U}}_{0}-\dot{U_{0}^{\prime }}\right|\ll \left|{\dot{U}}_{a}+{\dot{U}}_{0}\right|$, so the change in zero-sequence voltage can be ignored, and so Equation (6) can be simplified as follows:

(7) $$\Delta {\dot{I}}_{i}\approx \frac{\left({\dot{U}}_{a}+{\dot{U}}_{0}\right)}{R_{d}}$$

In the formula, $\left({\dot{U}}_{a}+{\dot{U}}_{0}\right)$ is the phase-to-ground voltage of the faulted phase A.

Similarly, the zero-sequence current difference before and after the fault of the non-fault line is as follows:

(8) $$\Delta \dot{I}=\frac{3({\dot{U}}_{0}-{\dot{U^{\prime }}}_{0})}{Z}$$

In practical engineering, since it is impossible to predict the time when the fault occurs, the vector of the zero-sequence current can be collected in real time, and the variation in the zero-sequence current can be obtained by the difference in the vector of the zero-sequence current collected at two different times, as shown in Equation (9):

(9) $$\Delta {\dot{I}}_{t}={\dot{I}}_{n\Delta t}-{\dot{I}}_{(n-2)\Delta t}$$

In the formula, ${\dot{I}}_{n\Delta t}$ is the zero-sequence current vector of the nth cycle, ${\dot{I}}_{(n-2)\Delta t}$ is the zero-sequence current vector of two cycles before the nth cycle, and $\Delta {\dot{I}}_{t}$ is the zero-sequence current variation in practical engineering.

### 2.2. Steady-State Impedance Analysis of High-Impedance Ground Faults in a Neutral-Point Grounding System with an Arc-Suppression Coil

**Figure 6:**
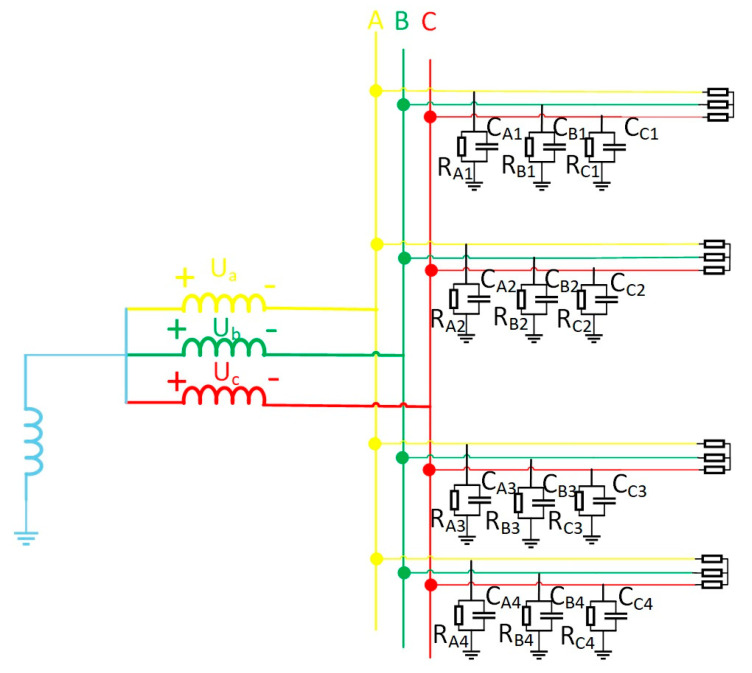
The equivalent model of arc-suppression-coil grounding.

Certain coal mine power grids use arc-suppression-coil grounding. While facilitating single-phase fault arc extinction, its compensation effect hampers precise faulted line identification via conventional methods [24]. This work thus analyzes arc-suppression-coil grounding circuits. Coal mine networks primarily operate in over-compensation mode, modeled in Figure 6.

**Figure 7:**
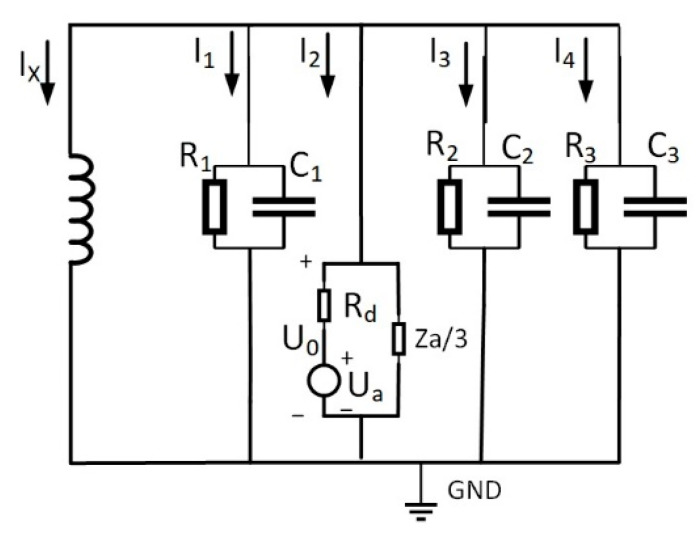
The zero-sequence equivalent model of arc-suppression-coil grounding.

The equivalent operational circuit for the fault is shown in Figure 4. The zero-sequence equivalent network of the arc-suppression-coil grounding system during a fault is shown in Figure 7.

The relationship between zero-sequence voltage and phase voltage in the neutral grounding fault model through an arc-suppression coil is shown in Equation (10):

(10) $${\dot{U}}_{0}=-\frac{{\dot{U}}_{a}}{(j\omega C_{\Sigma }-\frac{j}{\omega L}+\frac{1}{R_{\Sigma }})R_{d}}=-\frac{{\dot{U}}_{a}}{\sqrt{\frac{R_{d}^{2}}{R_{\Sigma }^{2}}+(\omega C_{\Sigma }R_{d}-\frac{R_{d}}{\omega L}{)}^{2}}}e^{-j\vartheta }$$

In the formula, L is the inductance of the grounding arc-suppression coil, $\vartheta =\arctan(j\omega C_{\Sigma }R_{\Sigma }-\frac{R_{\Sigma }}{\omega L})$, ϑ∈(−90° −0).

Comparing Equation (5) with Equation (10) reveals that variation in the system’s overall insulation parameters is influenced by the system’s neutral grounding method. Although the neutral grounding method affects the system’s zero-sequence voltage, it does not affect the relationship between the cable’s zero-sequence electrical signals and the insulation parameters.

Comparing Equation (7) with Equation (8) reveals the following: (1) fault line zero-sequence current variation exhibits distinct phase/magnitude characteristics versus that of non-fault lines, while non-fault lines share identical variations; (2) the fault line variation phase matches its faulty phase voltage.

Thus, fault line selection is achieved through the cross-line comparison of zero-sequence current variation phase and magnitude differences.

Coal mining environments yield microampere-scale line zero-sequence current changes. Practically, minute signals resist reliable recognition and incur high interference vulnerability. This work tackles this via a novel high-resistance grounding fault detection method merging steady-state impedance analysis with Holmes–Duffing oscillators. The approach extracts zero-sequence current features through impedance analysis, feeds them into noise-immune Holmes–Duffing systems for precise identification, and resolves minute fault signal recognition.

### 2.3. Adaptability Analysis of High-Impedance Grounding Fault Line Selection Criteria Based on SSIA

#### 2.3.1. High-Impedance Ground Fault Line Selection Criteria Considering Three-Phase Load Imbalance

In actual coal mine distribution networks, distribution network system terminals are interfaced with loads to energize them, leading to three-phase load imbalance conditions. To validate the applicability of the proposed fault line selection method under actual conditions, this section primarily examines the impact of three-phase load imbalance on the method.

**Figure 8:**
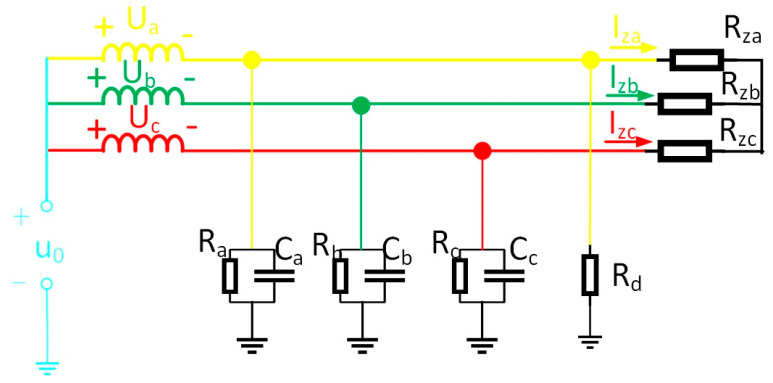
Equivalent circuit of cables with a three-phase unbalanced load.

Taking the single-phase high-resistance grounding fault as an example, the load adopts a Y-type connection, and the equivalent circuit of the system is shown in Figure 8. $R_{a},\;R_{b},\;R_{c}$ in the figure show the sizes of the three-phase load. When the three-phase load is unbalanced, the current flowing into the load will be asymmetrical, but according to Kirchhoff’s current law, the line current flowing into the load still meets ${\dot{I}}_{za}+{\dot{I}}_{zb}+{\dot{I}}_{zc}=0$.

Calculate the zero-sequence current according to Kirchhoff’s current law, as shown in Equation (11):

(11) $$\begin{array}{l} {\dot{Ι}}_{i}=\frac{\left({\dot{U}}_{a}+{\dot{U}}_{0}\right)}{Z_{a}//R_{d}}+\frac{\left({\dot{U}}_{b}+{\dot{U}}_{0}\right)}{Z_{b}}+\frac{\left({\dot{U}}_{c}+{\dot{U}}_{0}\right)}{Z_{c}}+{\dot{I}}_{za}+{\dot{I}}_{zb}+{\dot{I}}_{zc}\\ =\frac{\left({\dot{U}}_{a}+{\dot{U}}_{0}\right)}{Z_{a}//R_{d}}+\frac{\left({\dot{U}}_{b}+{\dot{U}}_{0}\right)}{Z_{b}}+\frac{\left({\dot{U}}_{c}+{\dot{U}}_{0}\right)}{Z_{c}} \end{array}$$

In the formula, ${\dot{I}}_{za},\;{\dot{I}}_{zb},\;{\dot{I}}_{zc}$ are the line currents flowing into the load.

Comparison of Equations (2) and (10) reveals identical expressions for zero-sequence current under both conditions. Even when three-phase imbalance occurs at the load terminal, it exerts no influence on the change quantity of zero-sequence current in each line before and after system faults. Analysis reveals that the presence of three-phase load imbalance in the system exerts no influence on the criteria proposed in this paper.

#### 2.3.2. High-Impedance Ground Fault Line Selection Criteria Considering the Influence of Different Cable Parameters

Actual coal mines exhibit diverse line configurations, with the cables employed varying across scenarios, leading to non-uniform parameters such as impedance, length, and distributed capacitance per line. Varying cable parameters may influence the criteria proposed herein. Hence, this section primarily addresses the impact of faults occurring on different lines on the proposed method.

The preceding analysis examined system zero-sequence components using Line 2 fault as an example. When faults occur on different lines, they exert no influence from a theoretical analysis perspective. Consequently, the criteria proposed in this paper remain valid.

## 3. High-Resistance Grounding Fault Identification Method Based on the Holmes–Duffing Oscillator System

This section focuses on the principles of the Holmes–Duffing oscillator system and the configuration of its parameters.

### 3.1. Small-Signal Detection Principle of the Holmes–Duffing Oscillator System

The Holmes–Duffing oscillator system mainly serves fundamental non-linear dynamics research. It features high parameter sensitivity and resists noise disturbances, processing perturbations while amplifying micro-signals [25]. Equation (12) confirms that proper parameter selection eliminates specific-frequency interference. When resonating, the system significantly amplifies micro-signals [26].

(12) $$\ddot{x}\left(t\right)\;+\delta \dot{x}\left(t\right)\;-x\left(t\right)\;+x^{3}\left(t\right)\;=p\cos\left(\omega t\right)$$

Write Equation (12) into a state as Equation (13):

(13) $$\left\{\begin{array}{ll} \dot{x}=y & \\ \dot{y}=-\delta y+x-x^{3}+p\cos(\omega t) & \end{array}\right.$$

In the formula, x is the displacement of the oscillator, δ is the damping coefficient, pcos(t) is the external driving force, p is the driving coefficient of the external force, and ω is the frequency of the external driving force. The Holmes–Duffing oscillator system alters its dynamics by adjusting the external driving force coefficient. Increasing this coefficient transitions the system through homoclinic orbits, period-doubling bifurcations, chaos, and periodic states [13].

### 3.2. Parameter Settings for Detection Based on the Holmes–Duffing Oscillator System

**Figure 9:**
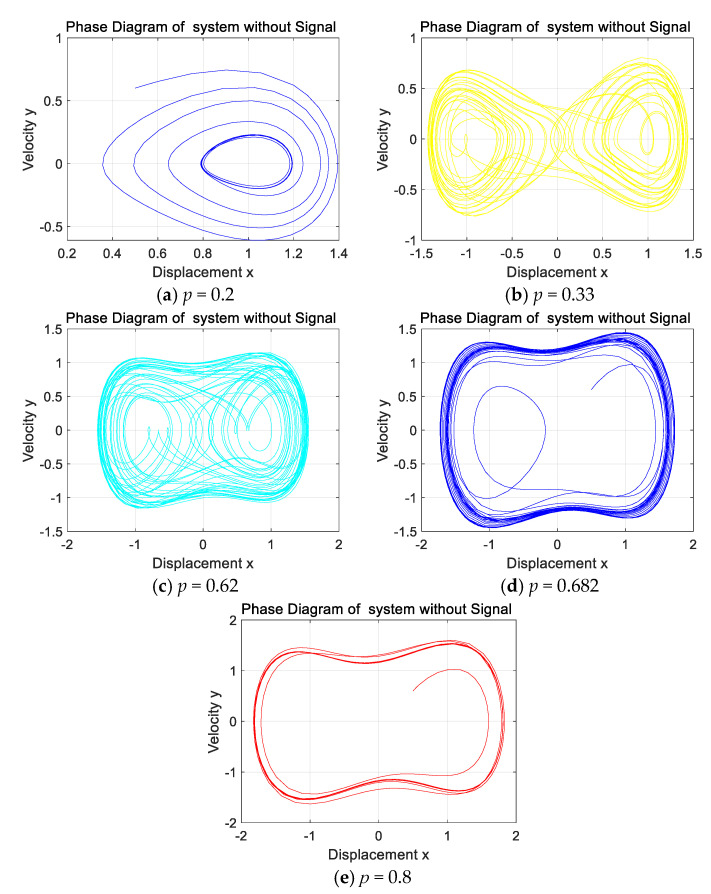
Phase diagram of the system output for different values of *p* without signal input.

When no signal is added to the system, the Holmes–Duffing oscillator system is simulated and the phase diagram in Figure 9 is obtained. Set the damping ratio δ=0.4, the initial displacement x=0.5, the initial velocity y=0.6, and the external driving force frequency ω=1. If the driving coefficient p increases from 0, the system will gradually show a homoclinic orbit, period-doubling bifurcation, a chaotic state, and a large periodic state [27]. In Figure 9, (a) is the homoclinic orbit, (b) is the period-doubling bifurcation state, (c) is the chaotic state, (d) is the critical state of the system from chaos to period, and (e) is the periodic state of the system [28].

By modulating its parameters, the Holmes–Duffing oscillator system is observed to exhibit profound non-linear characteristics [29]. When minute signals are input into the Holmes–Duffing oscillator system, despite minute distinctions between different signals, under the action of the system’s elevated sensitivity, distinct signals can be discriminated by analyzing output phase diagram states [30].

In coal mine fault line selection, the Holmes–Duffing system identifies faults by analyzing each line’s zero-sequence current changes. During high-resistance faults (Figure 1), it processes four-line current changes. Output phase diagrams show the following: faulted lines yield periodic states, non-faulted lines yield chaotic states (or vice versa). This distinct contrast provides reliable grounding fault selection criteria [14].

## 4. Algorithm Flow

This section presents the algorithm flowchart of this paper. To make the article’s logic clearer, algorithm pseudocode has been added to Appendix A, as shown in Algorithm A1.

Step 1: Collect the zero-sequence current of each line.

Step 2: Calculate the zero-sequence current vector of each line through FFT and calculate Equation (9).

Step 3: The variation in zero-sequence current of each line is obtained and used as the input of the Holmes–Duffing oscillator system.

Step 4: Calculate the Holmes–Duffing oscillator system algorithm to obtain the output phase diagram.

Step 5: Judging the output phase diagram, the line with the same phase diagram status is a healthy line, and the line with a different phase diagram status is a faulty line.

**Figure 10:**
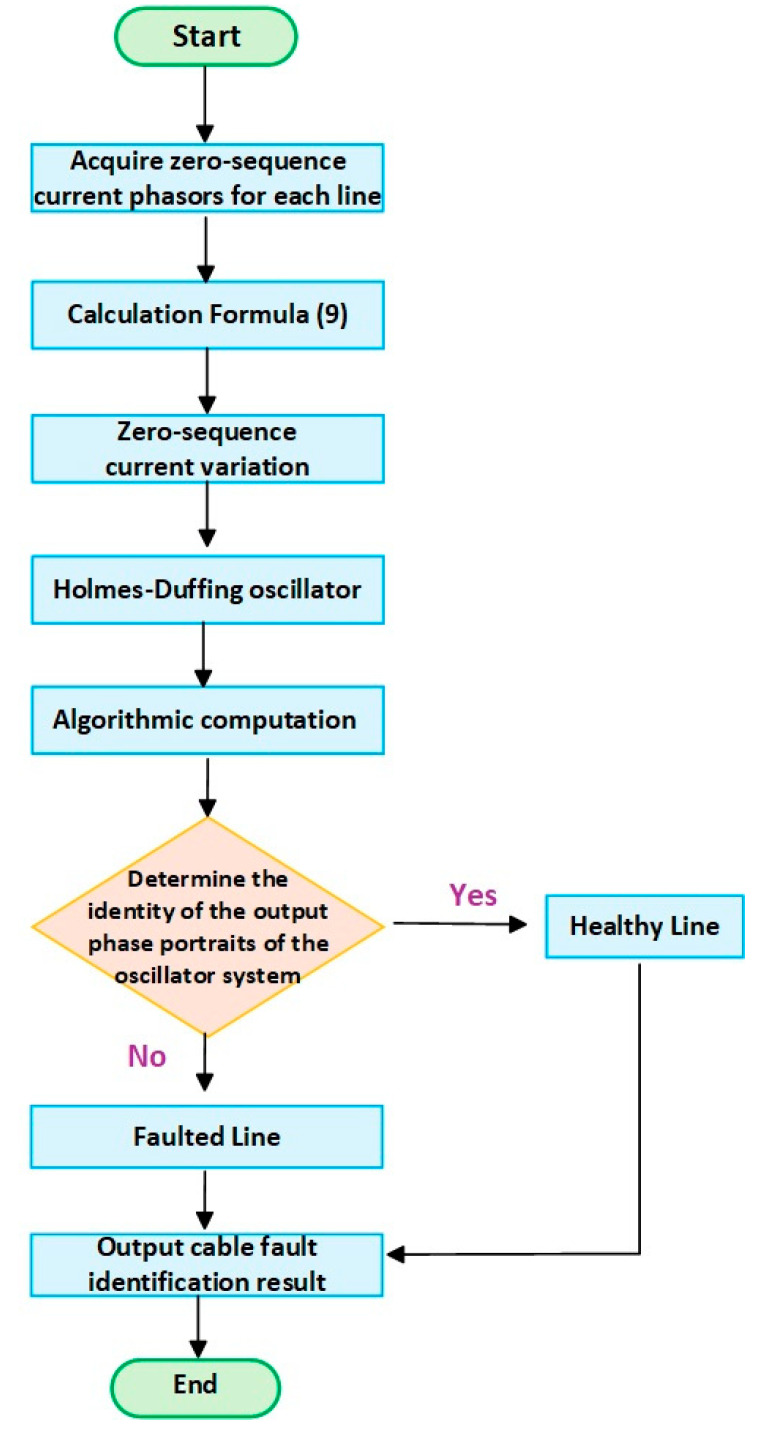
Algorithm flowchart.

The detailed flowchart is shown in Figure 10.

## 5. Calculus Analysis

This section is dedicated to validating the effectiveness of the criteria proposed in Section 2 under both ungrounded neutral and neutral grounded via arc-suppression-coil conditions, including scenarios with three-phase load unbalance and different cable parameters. Concurrently, it verifies the efficacy of the Holmes–Duffing oscillator system under the aforementioned operating conditions.

### 5.1. Simulation Model Building

**Figure 11:**
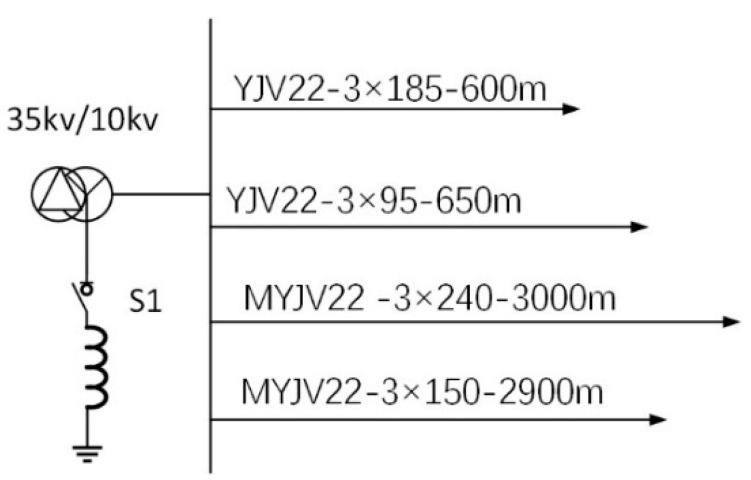
Cable parameter simulation model.

The simulation software employs MATLAB to validate the effectiveness of the proposed line selection strategy in the discussed scenarios. Aligned with actual coal mine conditions, the partial hardware is simplified, with the core section configured as the simulation model illustrated in Figure 11. The model delineates one main feeder and four outgoing lines under a 10 kV environment, alongside parameters of four cables [31]. A switch is positioned at the neutral point, enabling the realization of two grounding modes—ungrounded neutral and neutral grounded via an arc-suppression coil—through switch control.

By consulting the design and calculation manual of cable thermal characteristic parameters, the parameters of each line in the simulation model under normal operation can be calculated, as shown in Table 1.

Given the system parameters [32], set the damping coefficient to a fixed value of δ=0.4, the initial displacement x=0.5, the initial velocity y=0.6, the grounding impedance $R_{d}=90\;K\Omega$, then determine the parameters of the external driving force through experiments as p=0.6827, and set the modeling simulation frequency to f=50 Hz.

### 5.2. Effectiveness Analysis of High-Impedance Grounding Fault Line Selection Criteria Based on SSIA

#### 5.2.1. Ungrounded Neutral System

In Figure 11, switch S1 is open; the neutral point adopts an ungrounded mode. A fault is configured on Line 1, considering both three-phase balanced and unbalanced system load conditions. Zero-sequence currents of each line before and after the fault are acquired, respectively, differenced to derive the change quantities of the zero-sequence current, and their phase magnitudes are computed. The results under three-phase balance are tabulated in Table 2, while the results under three-phase imbalance are tabulated in Table 3.

A comparison of Table 2 and Table 3 delineates that the influence of three-phase load states on the change quantity of the zero-sequence current per line is negligible. This validates the adaptability of the criteria to load imbalance. Concurrently, the phase of the zero-sequence current change quantity on Line 1 exhibits opposition to that of the other three lines, identifying Line 1 as the faulted line. This outcome aligns with simulation settings, and phase consistency across the other lines corroborates the criteria’s effectiveness in ungrounded neutral systems.

#### 5.2.2. Neutral-Point Grounding via an Arc-Suppression-Coil System

The neutral point is grounded through an arc-suppression coil. Conduct the simulation as shown in the above section, and the results of the three-phase balance are shown in Table 4. The results of the three-phase unbalance are shown in Table 5.

An analysis of Table 4 and Table 5 delineates Line 1 as the faulted line, consistent with the simulation-configured faulted line. This corroborates the efficacy of the criteria under neutral grounding via an arc-suppression coil.

#### 5.2.3. Analysis of the Effect of Different Cable Parameters on the Criteria

This section explores the influence of different cable parameters on the criteria. The neutral point is ungrounded, and Line 3 (the longest line) is set to fault. The results are shown in Table 6. The neutral point is grounded through an arc-suppression coil, and Line 3 is a fault line. The results are shown in Table 7.

By analyzing Table 6 and Table 7, it is determined that Line 3 is a fault line, which is consistent with the fault line set by simulation.

### 5.3. High-Resistance Grounding Fault Line Selection and Identification Method Based on SSIA–Holmes–Duffing

#### 5.3.1. For Ungrounded Neutral Systems

**Figure 12:**
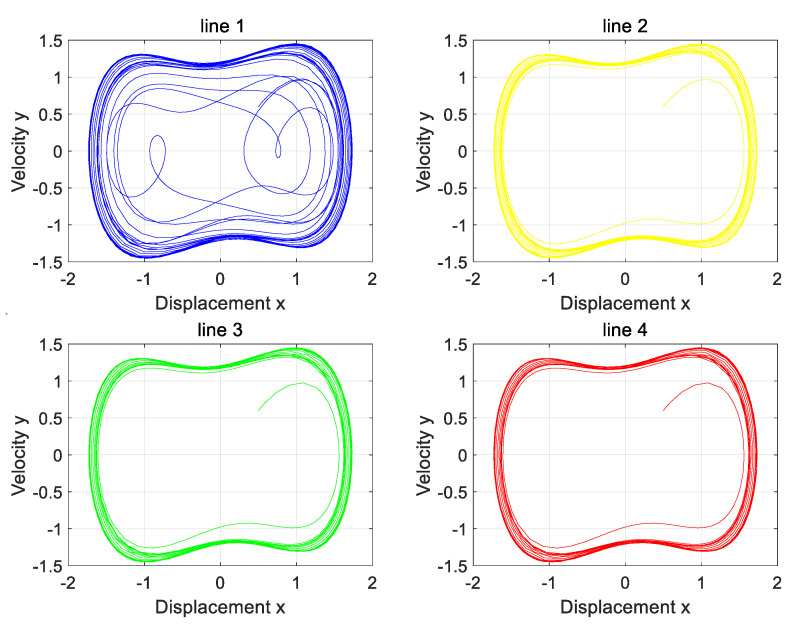
The phase diagram of the Holmes–Duffing oscillator system when the neutral point is not grounded.

The three-phase load is in a balanced state, both of which are 1000. At the same time, Line 1 is set to have a high-resistance grounding fault. The variation in zero-sequence current before and after the fault of four lines is input into the Holmes–Duffing oscillator system for simulation verification, and the simulation results as shown in Figure 12 are obtained.

Analysis of the four phase diagrams shows that Line 1’s output phase diagram is chaotic, while the others are periodic. Line 1’s diagram differs markedly from the other three. Per the theory in Section 2, Line 1 has a high-resistance grounding fault. This matches the simulation settings and aligns with the proposed strategy.

#### 5.3.2. For Neutral-Point Grounding via an Arc-Suppression-Coil System

**Figure 13:**
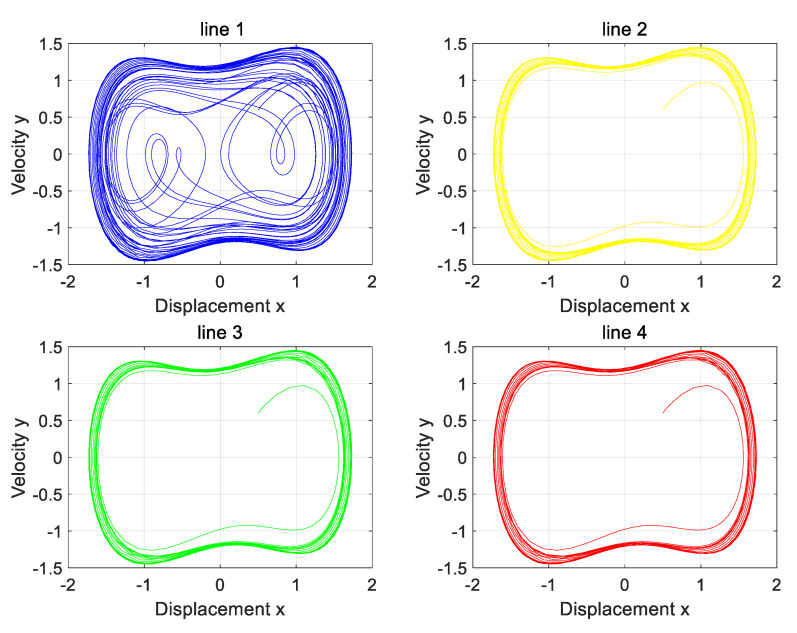
The phase diagram of the Holmes–Duffing oscillator system when the neutral point is grounded through an arc-suppression coil.

The neutral point is set to be grounded through the arc-suppression coil, and the over-compensation method is adopted. The over-compensation degree is generally in the range of 0–10%. According to $n=\frac{I_{L}-I_{C\sum }}{I_{C\sum }}\times 100\%$, the size of the grounding inductance can be calculated as 13.26H≤L≤14.6H. In this paper, the grounding inductance L=13.5H is taken for the simulation, and the output phase diagram is shown in Figure 13.

By comparing the four phase diagrams in Figure 13, it is found that Line 1 has a single-phase high-resistance grounding fault.

#### 5.3.3. Consider the Effect of the Presence of Three-Phase Load Imbalance in the System

**Figure 14:**
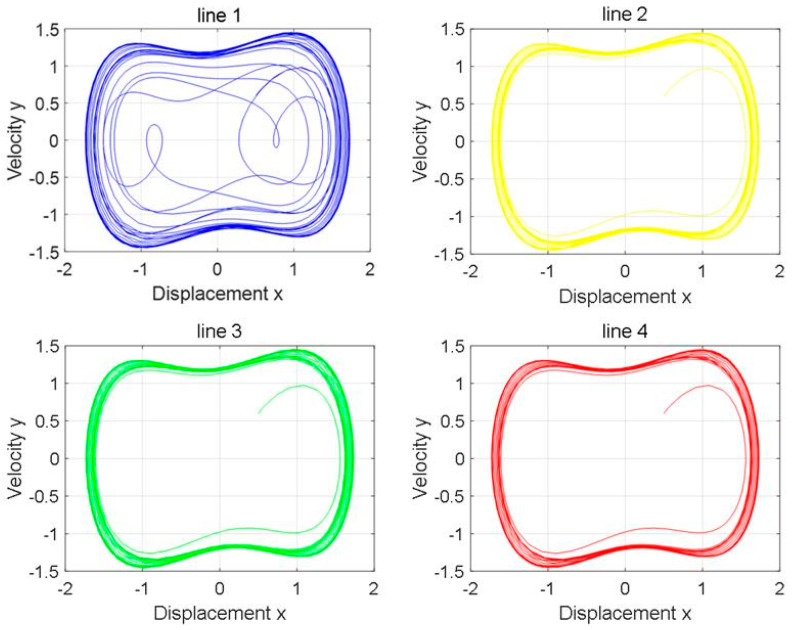
The phase diagram of the Holmes–Duffing oscillator system with an unbalanced load when the neutral point is not grounded.

**Figure 15:**
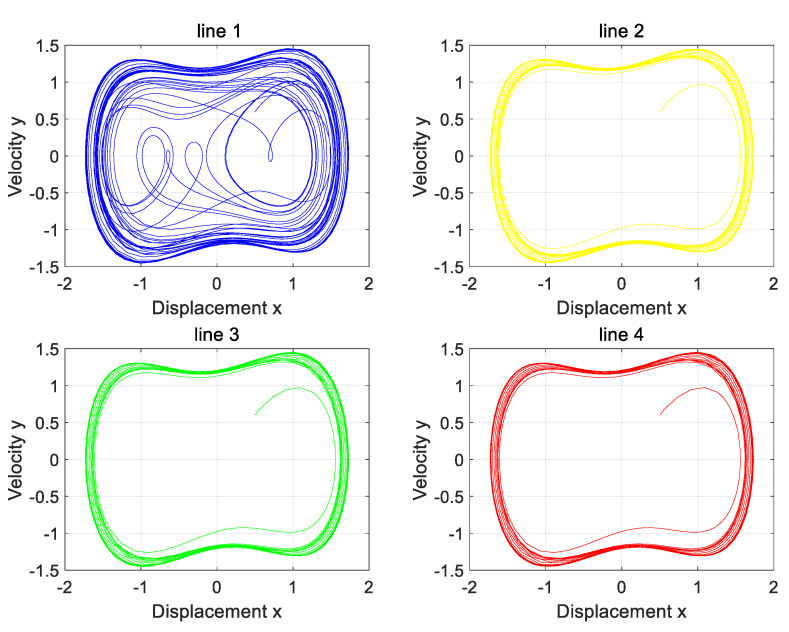
The phase diagram of the Holmes–Duffing oscillator system with an unbalanced load when the neutral point is grounded through an arc-suppression coil.

Previous derivations and theoretical analysis confirm that three-phase load imbalance exerts no impact on the proposed line selection strategy. This section validates these conclusions via simulation. Imbalanced load conditions are listed in Table 8. Simulations cover both ungrounded neutral and arc-suppression-coil grounding conditions. Output phase diagrams are shown in Figure 14 and Figure 15.

The phase diagram analysis shows that Line 1 is in a chaotic state, while the rest of the lines remain in a periodic state, and Line 1 is accurately identified as a faulty line. The results are in good agreement with the simulation settings, which verifies the effectiveness of the proposed line selection method under unbalanced load conditions.

#### 5.3.4. Consider the Effect of Different Cable Parameters

This section verifies the applicability of the line selection method to other line faults.

**Figure 16:**
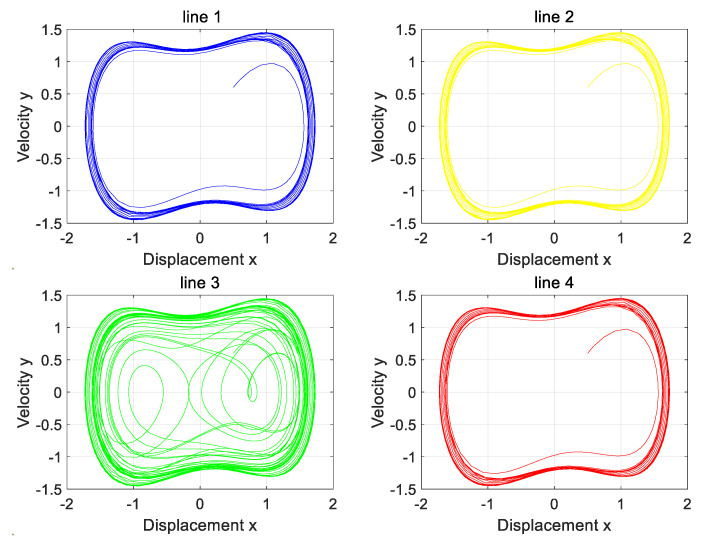
The phase diagram of the Holmes–Duffing oscillator system output when a fault occurs on the longest line with the neutral point ungrounded.

**Figure 17:**
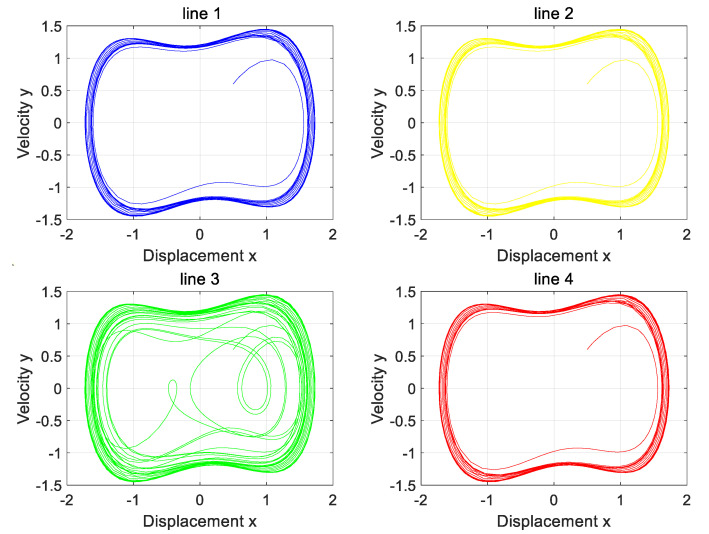
The phase diagram of the Holmes–Duffing oscillator system output when a fault occurs on the longest line with the neutral point grounded through a reactance coil.

Figure 16 and Figure 17 analysis shows that only Line 3 presents a chaotic state, and the rest of the lines maintain periodic states. The high-resistance grounding fault of Line 3 is accurately identified, which is completely consistent with the simulation settings, and verifies the effectiveness of this method.

## 6. Conclusions

This paper proposes a fault line selection and identification method for mine cables with non-effective grounding faults, based on the fusion of SSIA and Holmes–Duffing oscillator small-signal detection. Through verification with actual system case studies, the following conclusions are drawn: Through SSIA analysis, it can be seen that the zero-sequence current variation in the fault line is different from that of the non-fault line in phase and amplitude, and its phase is consistent with the fault phase to ground voltage. The above conclusions are verified by modeling and simulation. In this paper, a fault line selection method for mine cables based on SSIA and Holmes–Duffing oscillator detection is proposed. The method realizes fault identification by analyzing the phase diagram state generated by the input of zero-sequence current variation into the oscillator system. The effectiveness and applicability of the method are verified by a numerical example. Based on the fault line selection of non-effectively grounded systems, the influence of grounding impedance on the line selection criterion will be further considered in the future, and then the applicable grounding impedance range of this method will be determined.

## Figures and Tables

**Table 1:** Parameter values of each line in the simulation model.

	Line 1	Line 2	Line 3	Line 4
Capacitor (uf)	0.232	0.195	0.6	0.58
Inductor (mh)	0.157	0.201	0.0012	0.00116
Resistor (Ω)	0.0595	0.125	0.375	0.5974

**Table 2:** The phase change of the zero-sequence current in each line of a balanced three-phase load with an ungrounded neutral.

ΔI	Line 1	Line 2	Line 3	Line 4
phase	$-59.86^{\circ }$	$120.14^{\circ }$	$120.14^{\circ }$	$120.14^{\circ }$
amplitude	$3.385\times 10^{-3}$ A	$4.8\times 10^{-4}$ A	$1.477\times 10^{-3}$ A	$1.426\times 10^{-3}$ A

**Table 3:** The phase of the change in zero-sequence current in each line of an unbalanced three-phase load with an ungrounded neutral point.

ΔI	Line 1	Line 2	Line 3	Line 4
phase	$-59.86^{\circ }$	$120.14^{\circ }$	$120.14^{\circ }$	$120.14^{\circ }$
amplitude	$3.384\times 10^{-3}$ A	$4.799\times 10^{-4}$ A	$1.477\times 10^{-3}$ A	$1.428\times 10^{-3}$ A

**Table 4:** The phase change of the zero-sequence current in each line of a balanced three-phase load with the neutral grounded through a reactance grounding coil.

ΔI	Line 1	Line 2	Line 3	Line 4
phase	$-59.84^{\circ }$	$120.16^{\circ }$	$120.16^{\circ }$	$120.16^{\circ }$
amplitude	$3.338\times 10^{-3}$ A	$5.208\times 10^{-4}$ A	$1.603\times 10^{-3}$ A	$1.549\times 10^{-3}$ A

**Table 5:** The phase change of the zero-sequence current in each line of an unbalanced three-phase load with the neutral grounded through a reactance grounding coil.

ΔI	Line 1	Line 2	Line 3	Line 4
phase	$-59.84^{\circ }$	$120.16^{\circ }$	$120.16^{\circ }$	$120.16^{\circ }$
amplitude	$3.338\times 10^{-3}$ A	$5.207\times 10^{-4}$ A	$1.602\times 10^{-3}$ A	$1.549\times 10^{-3}$ A

**Table 6:** The phase change of the zero-sequence currents of each line in the longest line fault with an ungrounded neutral point.

ΔI	Line 1	Line 2	Line 3	Line 4
phase	$120.16^{\circ }$	$120.16^{\circ }$	$-59.84^{\circ }$	$120.16^{\circ }$
amplitude	$5.72\times 10^{-4}$ A	$4.8\times 10^{-4}$ A	$2.48\times 10^{-3}$ A	$1.428\times 10^{-3}$ A

**Table 7:** The phase change of the zero-sequence currents of each line in the longest line fault with the neutral point grounded through an arc-suppression coil.

ΔI	Line 1	Line 2	Line 3	Line 4
phase	$120.16^{\circ }$	$120.16^{\circ }$	$-59.84^{\circ }$	$120.16^{\circ }$
amplitude	$6.206\times 10^{-4}$ A	$5.208\times 10^{-4}$ A	$2.356\times 10^{-3}$ A	$1.549\times 10^{-3}$ A

**Table 8:** Load parameter values for each line.

Line	Line 1	Line 2	Line 3	Line 4
Phase A Ω	1000	950	1000	
Phase B Ω	1100	1000	1100	800
Phase C Ω	800	850	840	1000

## Data Availability

The original contributions presented in this study are included in the article. Further inquiries can be directed to the corresponding author(s).

## Appendix A

**Algorithm A1.** Holmes Duffing Chaotic System with Multi-Signal Analysis
1. Initialize System Parameters:
2. - Damping coefficient (delta) = 0.4
3. - Linear term coefficient (alpha) = −1
4. - Nonlinear term coefficient (beta) = 1
5. - External driving force amplitude (gamma) = 0.6827
6. - External driving force frequency (omega) = 1
7. Initialize Initial Conditions:
8. - Initial displacement (x0) = 0.5
9. - Initial velocity (x_dot0) = 0.6
10. - Initial state vector y0 = [x0; x_dot0]
11. Set Time Span:
12. - tspan = [0, 300]
13. Signal Processing for Four Lines:
14. for i = 1 to 4 do
15. Extract baseline signal: s_i0 = I_output_i0.signals.values
16. Extract measurement signal: s_i = I_output_i.signals.values
17. Calculate difference signal: s_i = s_i − s_i0
18. Generate time vector: t_s_i = linspace(tspan(1), tspan(2), length(s_i))
19. end for
20. Solve Duffing Equations with Signals:
21. for each signal i = 1 to 4 do
22. [t_signal_i, y_signal_i] = ode45(@holmes_duffing_with_signal_i, tspan, y0)
23. Extract displacement: x_signal_i = y_signal_i(:, 1)
24. Extract velocity: x_dot_signal_i = y_signal_i(:, 2)
25. end for
26. Phase Diagram Visualization:
27. for each line i = 1 to 4 do
28. Create new figure
29. Plot phase diagram: plot(x_signal_i, x_dot_signal_i)
30. Set title: "line i"
31. Set labels and formatting
32. end for
33. Function Definition—Duffing Equation with Signal:
34. function dydt = holmes_duffing_with_signal(t, y, parameters, signal_data)
35. x = y(1)
36. x_dot = y(2)
37. dxdt = x_dot
38. Interpolate signal: s_t = interp1(t_signal, signal, t, ‘linear’, ‘extrap’)
39. Calculate acceleration:
40. $\dot{y}(1)=x^{\prime }$
41. $\dot{y}(2)=-\delta x^{\prime }-\alpha x-\beta x^{3}+\gamma \cos(\omega t)+s(t)$
43. end function
44. Termination:
45. - All phase diagrams generated and displayed
46. return Analysis Results
